# The Effect of Guilingji Capsules on Vascular Mild Cognitive Impairment: A Randomized, Double-Blind, Controlled Trial

**DOI:** 10.1155/2022/4778163

**Published:** 2022-01-25

**Authors:** Huiqin Zhang, Hanqing Chen, Hui Pei, Huichan Wang, Lina Ma, Hao Li

**Affiliations:** ^1^Department of Geriatrics, Xiyuan Hospital, China Academy of Chinese Medical Sciences, Beijing 100091, China; ^2^First Clinical College, Shandong University of Traditional Chinese Medicine, Jinan, Shandong 250355, China

## Abstract

Guilingji capsules (GLJC) have been shown to have antiaging effects and improve cognitive function. The aim of this study was to evaluate the clinical efficacy and safety of GLJC for the treatment of vascular mild cognitive impairment (VaMCI). A total of 96 patients with VaMCI (aged 60–85 years) were enrolled in this 24-week, randomized, double-blind, controlled clinical trial. The patients were randomly assigned to a GLJC group (*n* = 48) or a Ginkgo group (*n* = 48). Patients in the GLJC group were treated using GLJC, whereas those in the Ginkgo group received Ginkgo extract tablets. We evaluated the participants at baseline and after a 12- and 24-week treatment period using the Montreal Cognitive Assessment (MoCA), Mini-Mental State Examination (MMSE), Alzheimer's Disease Assessment Scale-Cognitive Subscale (ADAS-Cog), and Chinese Medicine Symptom Scale (CM-SS). The serum acetylcholine (Ach), acetylcholinesterase (AchE), homocysteine (Hcy), and high-sensitivity C-reactive protein (hs-CRP) serum levels of the patients were measured before and after 24-week treatment. Analysis of the results of both groups showed that both interventions significantly increased the MoCA and MMSE scores of the patients and decreased their ADAS-Cog and CM-SS scores (*P* < 0.05). The GLJC group showed greater improvement in MoCA, MMSE, and CM-SS scores than the Ginkgo group (*P* < 0.05). However, both groups showed a significant increase in serum Ach and a decrease in serum AchE, Hcy, and hs-CRP levels (*P* < 0.05). Furthermore, serum Ach increased and Hcy decreased more significantly in the GLJC group than in the Ginkgo group (*P* < 0.05). These findings indicate that GLJC can improve the cognitive function, cholinergic system, and inflammatory cytokine levels of patients with VaMCI. Furthermore, this treatment can improve symptoms of syndromes diagnosed according to traditional Chinese medicine practice in patients with VaMCI.

## 1. Introduction

Vascular mild cognitive impairment (VaMCI) refers to mild cognitive impairment (MCI) syndrome caused by or related to various vascular factors. It is regarded as a potentially transitional condition between normal aging and vascular dementia (VaD) [1–3]. The global prevalence of MCI and VaD has increased concurrently with the rapid aging of the global population [4, 5]. Epidemiologic studies have reported that more than 6.7% of persons aged over 60 years have MCI and that the annual rate of its progression to dementia is growing [6, 7]. MCI is the early manifestation of VaD. However, VaMCI is considered an early stage of vascular cognitive impairment (VaCI) and accounts for the highest proportion of VaCI cases [1, 2]. Approximately 10% of patients with VaMCI progress to VaD every year, 19% progress to VaD after two years, and 46% progress to VaD after five years. Currently, the prevalence of VaMCI is estimated to be twice that of VaD [8, 9]. If intervention for VaMCI is not initiated on time, the risk for VaD, the second most common subtype of dementia, increases [10, 11]. Fortunately, VaMCI has a certain degree of reversibility and several controllable conditions related to vascular factors [12, 13]. Effective treatment of VaMCI is essential as it may protect patients from developing VaD and decrease the incidence of VaD [14–16].

VaMCI is a complex, multifactorial clinical syndrome of cognitive decline, which is affected by many factors [17–19]. For example, the cholinergic system plays a primary role in VaMCI [20]. Acetylcholine (Ach), an important neurotransmitter in the central cholinergic system, can regulate learning and memory function, which are closely related to cognitive function and VaCI. Thus, a decrease in Ach levels will lead to cognitive decline [21]. Acetylcholinesterase (AchE), a key enzyme found in cholinergic synapses, can degrade Ach and impair cognitive function [22, 23]. In addition, neuroinflammation and white matter pathology are risk factors for cerebrovascular disease, which is closely associated with VaMCI [24, 25]. Both high-sensitivity C-reactive protein (hs-CRP) and homocysteine (Hcy) can contribute to the inflammatory response and oxidative stress, damage vascular endothelium, and impair cognitive function, all of which play certain roles in the pathogenesis of VaMCI [26–29]. Valid therapies for the prevention of the progressive cognitive decline associated with VaMCI are not yet available [30]. In addition, the efficacy of anticholinergic drugs and glutamate receptor antagonists for the treatment of cognitive impairment is unclear [7, 30, 31].

Recently, many studies have indicated that herbal preparations (e.g., Ginkgo extracts and ginseng products) can improve learning and memory function in elderly patients with cognitive dysfunction and/or VaD [32–35]. As VaMCI is the early stage of VaD, traditional Chinese medicine (TCM) may have its own unique advantages for the treatment of VaMCI. In addition, herbal medicines are mainly characterized as safe to use with few side effects, and with multiple components and targets. Thus, herbal medicine serves as a good resource for discovering drugs for the treatment of VaMCI.

VaMCI is characterized by cognitive decline and is caused by small vessel disease, lacunar infarcts, and white matter changes [36]. In TCM, VaMCI is considered to be closely related to the brain and kidney. Therefore, the aim of TCM treatment for VaMCI is to tonify the kidney and essence, activate blood circulation, remove phlegm, and improve blood stasis. Guilingji, a classic TCM prescription that is recorded in the 2015 Chinese Pharmacopoeia, mainly consists of traditional Chinese herbs, including Ginseng Radix et Rhizoma Rubra. It is famous for its efficacy in strengthening the brain and kidney and is associated with lasting good health and longevity [37, 38]. Studies have also shown that Guilingji capsules (GLJC) possess significant antiaging effects and could extend the lifespan [39, 40]. Moreover, the results of animal experiments indicate that GLJC could improve learning and memory dysfunction in naturally aging rats [41, 42]. However, previous studies on GLJC mainly constitute animal studies [37, 38, 42] and clinical research on GLJC is limited [40]. Considering the antiaging effects of GLJC and its tonifying effect on the kidney and brain, appropriate research is needed to further verify its efficacy. Therefore, we conducted a clinical study to investigate the efficacy of GLJC for the treatment of cognitive decline in patients with VaMCI.

Previous studies have demonstrated that Ginkgo extracts can improve cognitive function and have certain curative effects on cognitive impairment and VaD [35, 43–45]. Furthermore, it can enhance the integrity of the cerebral microvasculature and strengthen the ability of cerebral microvascular endothelial cells to resist damage caused by hypoxia and amyloid-beta (A*β*) protein [46, 47]. Similarly, Ginkgo extract tablets have been reported to increase cerebral blood flow, improve hemodynamics, and protect brain function in patients with VaCI [43, 45]. Therefore, we selected Ginkgo extract tablets as a positive control treatment for the evaluation of the clinical efficacy and safety of GLJC for the improvement of VaMCI.

The Montreal Cognitive Assessment (MoCA) scale is mainly used to evaluate the perception of visual space, executive function, memory, attention, and language in patients with cognitive impairment. The MoCA scale has a higher sensitivity and credibility for MCI than the Mini-Mental State Examination (MMSE) scale [48]. Thus, the primary outcome of this study was the change in the MoCA scale score after intervention, whereas the secondary outcomes were changes in the MMSE, Alzheimer's Disease Assessment Scale-Cognitive Subscale (ADAS-Cog), and Chinese Medicine Symptom Scale (CM-SS) scores, as well as changes in serum indexes levels. In this study, we hypothesized that GLJC could improve cognitive decline in patients with VaMCI.

## 2. Materials and Methods

### 2.1. Trial Design

This was a 24-week, randomized, double-blind, controlled trial conducted to evaluate the clinical efficacy of GLJC. This study was performed at the outpatient and inpatient departments of the Xiyuan Hospital of China Academy of Chinese Medical Sciences and the Affiliated Hospital of Shandong University of Traditional Chinese Medicine from August 2019 to October 2020. The study protocol was approved by the Ethics Committee of Xiyuan Hospital, China Academy of Chinese Medical Sciences (approval number: 2018XLA039-2). An external data and safety monitoring committee was formed for this study, and the trial was registered with the China Clinical Trials Registry (ID: https://clinicaltrials.gov/ct2/show/NCT03647384) on August 23, 2018.

### 2.2. Participants

The inclusion criteria for this study were as follows: (1) age between 60 and 85 years; (2) fulfillment of the diagnostic criteria for VaMCI according to the fifth edition of the Diagnostic and Statistical Manual of Mental Disorders [49] and the guidelines of the American Heart Association/American Stroke Association [50]; (3) brain magnetic resonance imaging and computed tomography (CT) scans consistent with a diagnosis of MCI [3]; (4) Clinical Dementia Rating (CDR) score ≤0.5 [51]; (5) MMSE score >24 and MoCA score >22, indicating a diagnosis of MCI and not VaD; (6) case history that indicates a causal relationship between cognitive decline and cerebrovascular disease, for example, cognitive impairment that occurred within 3 months of cerebrovascular disease, Hachinski Ischemic Scale (HIS) score ≥7, or absence of cerebrovascular disease but presence of a decrease in information processing and/or execution function; and (7) Activity of Daily Living (ADL) score ≤26.

The exclusion criteria were as follows: (1) severe neurological deficits, such as Parkinson's disease, epilepsy, brain tumors, brain edema, traumatic brain injuries, hypothyroidism, encephalitis, Huntington's disease, severe anemia, infections, or other endocrine diseases; (2) depression (reference, Hamilton Depression Rating Scale) or other mental diseases; (3) recent use (within 3 months) of drugs that may affect cognitive function or an allergy to the drugs used in this study; and (4) an allergy to Ginkgo extract tablets and Chinese medicine ingredients.

### 2.3. Randomization and Blinding

SAS 9.2 software (SAS Institute Inc., Cary, NC, USA) was used to generate random numbers. The random numbers were assigned to the participants by an independent statistician at the GCP Center of Xiyuan Hospital. All participants were randomly and evenly divided either into the Ginkgo group (*n* = 48) or the GLJC group (*n* = 48) using blocked randomization (block = 16, length = 6). Drugs were numerically labeled and sequenced according to the random numbers, and the patients were assigned to either group in accordance with the sequence. Patients received either Ginkgo extract tablets and a placebo or GLJC and a placebo, depending on their group. The clinicians, participants, statistician, and outcome assessors were blinded to the assignment until completion of the study. Allocation concealment was performed using sealed opaque envelopes. The database was locked after the input of all data, and group information were revealed when statistical analyses were conducted. Unblinding was allowed only in the case of a patient emergency.

### 2.4. Intervention

Patients in the GLJC group took Guilingji capsules (0.6 g, orally, once daily; provided by Guangyuyuan Co., Ltd., Shanxi, China; Batch No.: 103180601) and placebo Ginkgo extract tablets (19.2 mg, orally, three times daily). Patients in the Ginkgo group took Ginkgo extract tablets (19.2 mg, orally, three times daily; provided by Yangzijiang Pharmaceutical Group Co., Ltd., Nanjing, China; Batch No.: 10010321) combined with a placebo Guilingji capsule (0.6 g, orally, once daily). The placebo was consistent with the experimental drug in appearance, smell, and packaging. A 12-week intervention period constituted a course of treatment. Thus, the 24-week intervention period comprised two courses of treatment.

### 2.5. Serum Analyses

The blood samples of patients were obtained at baseline and at the endpoint of the study. After centrifugation, the upper serum layer was transferred to a disposable Eppendorf tube and frozen at −80°C until required. The serum samples were subsequently thawed at room temperature prior to analysis.

The serum indexes levels were measured at the laboratory of Xiyuan Hospital. Serum Ach and AchE levels were measured using an Ach assay kit (ab65345, Abcam, Cambridge, UK) and an AChE assay kit (ab138871, Abcam, Cambridge, UK), respectively. Serum Hcy levels were measured using an enzymatic method using a Roche Cobas e701 module (Roche Diagnostics GmbH, Mannheim, Germany). Serum hs-CRP levels were detected using the enzyme-linked immunosorbent assay method with an hs-CRP assay kit (HK369, Hycult Biotech). All the above-mentioned protocols were conducted according to the manufacturers' instructions.

### 2.6. Outcomes

Specially trained researchers assessed the MoCA, MMSE, ADAS-Cog, and CM-SS scores of the patients prior to intervention after 12 weeks and after 24 weeks. Serum Ach, AchE, Hcy, and hs-CRP levels were measured at baseline and at 24 weeks.

The primary outcome was the MoCA score. The change in MoCA score before and after the intervention was used to evaluate the efficacy of GLJC for improving cognitive impairment.

The secondary outcomes were the MMSE, ADAS-Cog, and CM-SS scale scores and serum Ach, AchE, Hcy, and hs-CRP levels. The MMSE is used worldwide to assess cognitive function, with higher MMSE scores indicating better cognitive function. It is used to assess orientation ability (time orientation, spatial orientation, and calculation), memory (immediate memory and delayed memory), linguistic ability (naming, comprehension, and retelling), comprehension, attention, and calculation ability [52, 53]. The ADAS-Cog is widely used to detect mild-to-moderate cognitive impairment. It includes 12 items, which are more sensitive than the MMSE in terms of the assessment of word recall and recognition, language understanding, and intentional practice. A higher score indicates a greater degree of cognitive impairment [54]. Changes in the MMSE and ADAS-Cog scores before and after intervention were used to evaluate the efficacy of GLJC in improving cognitive function. Regarding the CM-SS score, the following evaluation criteria were used to assess the clinical efficacy rate: efficacy index = (pre-treatment integral − post-treatment integral)/pre-treatment score × 100%. An efficacy index ≥95% indicates a cured case; ≥70% indicates efficacy; ≥30% to <70% indicates an improved case; and <30% indicates inefficacy. Efficacy rate = cured + effectiveness + improved rate.

The safety-related outcomes of this study were nausea/vomiting and allergic reactions that disappeared soon after discontinuation of the supplements. Furthermore, the occurrence of any adverse events during the treatment course was recorded.

### 2.7. Follow-Up

The participants were examined every 12 weeks; the course of the intervention lasted 24 weeks. In total, 14 patients dropped out during the study and 82 completed the trial. Among these, 37 patients were in the GLJC group and 45 in the Ginkgo group.

### 2.8. Statistical Analysis

SPSS software, version 25, was used to perform all statistical analyses. Normally distributed measurement data were analyzed using the independent samples *t*-test and are reported as mean ± standard deviation (SD). Non-normally distributed data were analyzed using a nonparametric test (the Wilcoxon test). Descriptive statistics are reported as median and interquartile range (IQR). Due to presence of non-normally distributed data, the Mann–Whitney *U* test was used for intergroup comparison, whereas the Wilcoxon signed-rank test was used for intragroup comparison. Continuous variables were analyzed using the chi-square test (*χ*^2^ test), and categorical variables were analyzed using the Ridit test. Statistical significance was set at *P* < 0.05.

## 3. Results

### 3.1. Study Population

**Figure 1:**
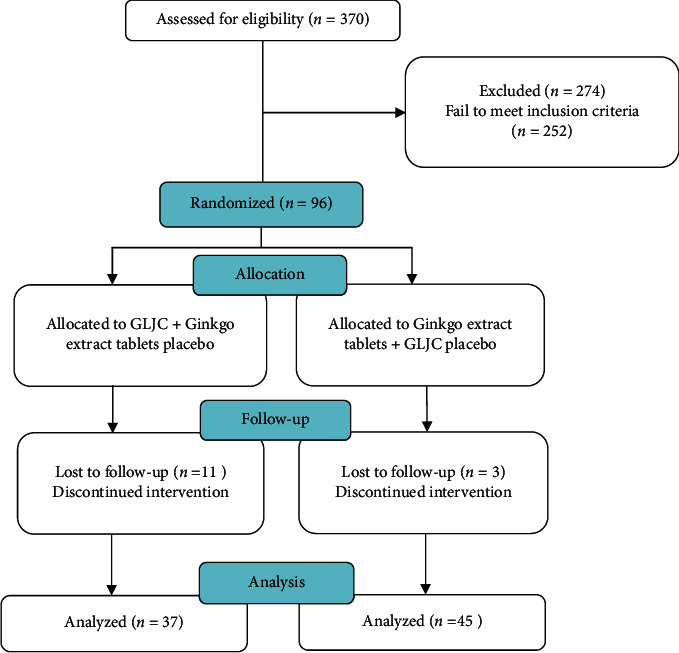
The CONSORT flowchart of the study.

We included 82 patients with VaMCI who completed the trial in the final analysis (Figure 1). Among these, 38 (46.34%) were male and 44 (53.66%) were female, with ages ranging from 60 to 85 years (mean: 70.06 ± 7.16 years). The Ginkgo group comprised 45 patients (18 male, 27 female) with a mean age of 70.58 ± 7.22 years. In the Ginkgo group, 15 patients had hypertension, 9 had diabetes, 11 had coronary heart disease, 8 had hyperlipidemia, 31 had cerebral infarction, 7 had cerebral ischemia, and 8 had leukoencephalopathy. The GLJC group comprised 37 patients (20 male, 27 female) with a mean age of 69.54 ± 7.10 years. In this group, 16 patients had hypertension, 12 had diabetes, 11 had coronary heart disease, 4 had hyperlipidemia, 21 had cerebral infarction, 9 had cerebral ischemia, and 10 patients had leukoencephalopathy. There were no significant differences in baseline data between the two groups (*P* > 0.05, Table 1).

### 3.2. Comparison of Preintervention Efficacy Indicators

The included 82 patients completed the MoCA, MMSE, ADAS-Cog, CM-SS, HIS, ADL, and CDR scales before treatment. The serum Ach, AchE, Hcy, and hs-CRP levels of the patients were also measured prior to the intervention. There were no significant differences in scale scores or serum indexes between the two groups (*P* > 0.05, Table 2).

### 3.3. Comparison of the MoCA, MMSE, and ADAS-Cog Scores after Intervention

#### 3.3.1. Comparison of Total MoCA, MMSE, and ADAS-Cog Scale Scores after the Intervention

**Figure 2:**
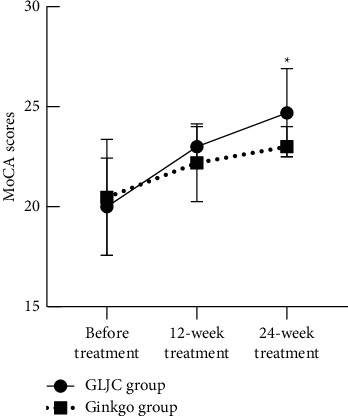
Comparison of MoCA scores in the 2 groups. Note: ^*∗*^significant effect (*P* < 0.05).

**Figure 3:**
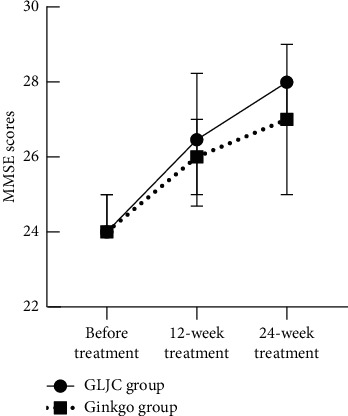
Comparison of MMSE scores in the 2 groups. Note: ^*∗*^significant effect (*P* < 0.05).

**Figure 4:**
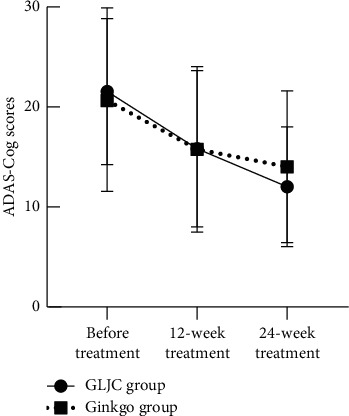
Comparison of ADAS-Cog scores in the 2 groups. Note: ^*∗*^significant effect (*P* < 0.05).

In the GLJC group, the MoCA score increased by 3 (1, 5) points, the MMSE score increased by 1 (1, 3) point, and the ADAS-Cog score decreased by 5 (2.35, 8) points after the 12-week treatment (*P* < 0.05). In the Ginkgo group, the MoCA score increased by 1 (1, 3) point, MMSE score increased by 2 (0, 3) points, and the ADAS-Cog score decreased by 4.87 ± 4 points after the 12-week treatment (*P* < 0.05). However, the differences in MoCA, MMSE, and ADAS-Cog scores between the two groups were not significant (*P* > 0.05). After the 24-week treatment, the MoCA score increased by 4.70 ± 3.17 points, MMSE score increased by 3 (1, 4) points, and the ADAS-Cog score decreased by 9.52 ± 3.97 points in the GLJC group (*P* < 0.05), whereas the MoCA score increased by 3 (0, 4) points, the MMSE score increased by 2 (1, 3) points, and the ADAS-Cog score decreased by 6.61 ± 4.09 points in the Ginkgo group (*P* < 0.05). The total MoCA and MMSE scale scores were higher in the GLJC group than in the Ginkgo group after the 24-week treatment (*P* < 0.05). However, the total ADAS-Cog scores were not significantly different between the two groups (*P* > 0.05) (Table 3, Figures 2–4).

#### 3.3.2. Comparison of Single Item Scores in the MoCA Scale after the 24-Week Treatment

The items of the MoCA scale are as follows: “visual space and execution,” “naming,” “attention,” “language,” “abstraction,” “delayed recall,” and “orientation.” There was a significant post-treatment improvement in the “visual space and execution,” “attention,” “abstraction,” “delayed recall,” and “orientation” items in the GLJC group (*P* < 0.05), whereas the Ginkgo group showed an improvement in the “visual space and execution,” “attention,” and “language” items (*P* < 0.05). However, “visual space and execution” and “orientation” scores increased more significantly in the GLJC group than in the Ginkgo group (*P* < 0.05, Table 4).

### 3.4. Comparison of Serum Ach, AchE, Hcy, and hs-CRP Levels after the 24-Week Treatment

Serum Ach levels increased in the GLJC and Ginkgo groups after the 24-week treatment (*P* < 0.05). However, serum Ach levels increased more significantly in the GLJC group than in the Ginkgo group (*P* < 0.05). Serum AchE, Hcy, and hs-CRP levels decreased in both groups after treatment (*P* < 0.05). However, serum Hcy levels decreased more significantly in the GLJC group than in the Ginkgo group (*P* < 0.05). Serum AchE and hs-CRP levels were not significantly different between the two groups after treatment (*P* > 0.05, Table 5).

### 3.5. Comparison of Pretreatment and Posttreatment CM-SS Scores

#### 3.5.1. Comparison of the Total CM-SS Scores

In the GLJC group, the total CM-SS score decreased by 4 (3–7) points after the 12-week treatment and decreased by 7.89 ± 4.28 points after the 24-week treatment (both *P* < 0.05). In the Ginkgo group, the total CM-SS score decreased by 2 (1–3) points after the 12-week treatment and decreased by 5.29 ± 2.78 points after the 24-week treatment (both *P* < 0.05). The total CM-SS scores after the 12-week and 24-week treatment periods were lower in the GLJC group than in the Ginkgo group (*P* < 0.05, Table 6).

#### 3.5.2. Comparison of the Overall Efficacy Rate of CM-SS after the 24-Week Treatment

The overall *efficacy* rate of symptoms in the CM-SS was 67.57% in the GLJC group and 46.67% in the Ginkgo group after the 24-week treatment. There was no statistically significant difference between the two groups (*P* > 0.05, Table 7).

#### 3.5.3. Comparison of the Efficacy of a Single Item in the CM-SS Scale after Treatment

**Figure 5:**
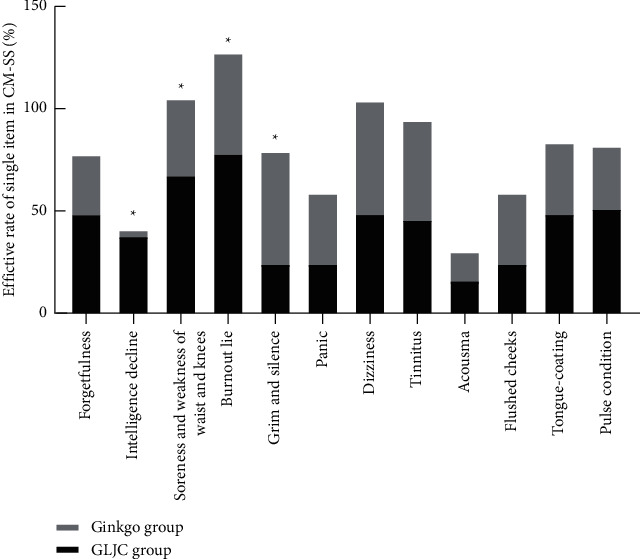
Comparison of efficiency of a single item in CM-SS in the 2 groups after treatment. Note: ^*∗*^significant effect (*P* < 0.05).

The efficacy of a single item in the CM-SS was evaluated using the nimodipine method. The efficacy of “intelligence decline,” “soreness and weakness of the waist and knees,” and “burnout-like” symptoms after the 24-week treatment was higher in the GLJC group than in the Ginkgo group (*P* < 0.05). Improvement in the “grim and silence” symptom after the 24-week treatment period was more significant in the Ginkgo group than in the GLJC group (*P* < 0.05). There were no significant differences in other single items of the CM-SS scale between the two groups (*P* > 0.05, Table 8, Figure 5).

### 3.6. Safety Evaluation

All safety, hematological, and biochemical parameters were within normal limits in the two groups at the end of the interventions. No adverse reactions occurred during this trial.

## 4. Discussion

In this study, we investigated the clinical efficacy and safety of GLJC for the treatment of patients with VaMCI using associated cognitive scales, the CM-SS, and related serum indicators. We found that GLJC had curative effects on VaMCI and improved cognitive function, the cholinergic system, and inflammatory reactions. Our findings also indicate that this treatment is safe.

The pathogenesis of VaMCI is complex, and no unified conclusion on its pathological process has been reached yet. The most important postulated pathogenesis includes white matter injury (WMI), cerebral infarction, and cerebral hemorrhage, which lead to the destruction of the blood-brain barrier and the neurovascular unit. The inflammatory response and oxidative stress lead to the release of a variety of growth factors and chemicals, which cause an increase in vascular permeability, protein exudation, blood-brain barrier impairment, and axonal demyelination, resulting in brain tissue damage and cognitive dysfunction [24, 55]. In addition, cerebral small vessel disease, which can damage the connection between the thalamus and cerebral cortex and striatal pathway, leading to cognitive dysfunction (mainly a decline in executive function), is also closely related to the occurrence of VaMCI [56]. An animal study demonstrated that Guilingji could improve the cognitive function of natural aging rats [42]. In TCM, GLJC, which mainly consists of Ginseng Radix et Rhizoma Rubra, has been reported to tonify the kidney and essence, strengthen the body and brain, and alleviate fatigue [39, 40]. Modern pharmacological studies have also shown that GLJC can improve memory, regulate neurotransmitter metabolism, delay the aging of monoamine neurons in the brain, and protect the neurons. Additionally, it is relatively safe to use [57, 58].

Studies have demonstrated that ginsenosides, the main components of ginseng, have therapeutic effects on cognitive impairment [35, 59, 60]. To some extent, ginsenoside Rb improves learning and memory ability through antioxidative stress, inhibits apoptosis, and promotes the release of Ach [61, 62]. Ginsenoside Re mainly regulates the function of the central cholinergic system by inhibiting AchE activity in the brain and plays a role in protecting neurons and improving cerebral ischemia and hypoxia, which can improve cognitive function and learning skills [33]. Ginsenoside Rg and ginsenoside Rh can upregulate the content of Ach in the brain by promoting the synthesis and release of neurotransmitters to delay aging and prevent the development of MCI [59, 60, 63]. Hence, these characteristics of the effective ingredients of GLJC may underlie the positive effects noted in patients with VaMCI.

The MoCA, MMSE, and ADAS-Cog scales were used to assess cognitive function in the present study. The MoCA scale has a higher sensitivity and credibility for MCI than the MMSE scale [48]. The MMSE scale is used worldwide to assess cognitive function, with higher MMSE scores indicating better cognitive function. However, it is susceptible to be affected by the respondent's educational level and lacks sensitivity for MCI that does not meet the diagnostic criteria for dementia; thus, it is often combined with the MoCA scale [52, 53]. The ADAS-Cog scale is more sensitive than the MMSE in terms of word recall and recognition, language understanding, and intentional practice, with higher ADAS-Cog scores indicating a greater degree of cognitive impairment [54]. In this study, the GLJC group showed a more significant improvement in the “visual space and execution” and “orientation” items of the MoCA scale and higher MMSE scores after treatment than the Ginkgo group. This result demonstrates that GLJC could improve cognitive function in patients with VaMCI.

The cholinergic system plays an important role in cognitive function, such as memory, learning, and attention [20]. Ach is an important neurotransmitter of the cholinergic system, which can stimulate the brain network to maintain brain arousal and regulate learning and memory. Thus, a loss of Ach will cause cognitive impairment [64]. AchE is a key enzyme that exists in cholinergic synapses, and it participates in the hydrolysis of Ach and can prevent the excitability of the postsynaptic membrane caused by neurotransmitters [22]. An increase in the serum AchE level is related to cognitive impairment [23, 65]. In the present study, we observed a significant post-treatment increase in serum Ach levels and a decrease in serum AchE levels in the GLJC group. This result indicates that GLJC could improve cognitive function by improving the cholinergic system, a finding which is consistent with those of previous studies that demonstrated that GLJC has antiaging effects [39–41, 66].

Cerebrovascular disease is an important risk factor of VaCI [24, 25]. Both Hcy and hs-CRP can mediate oxidation and inflammatory reactions, contributing to the release of a large amount of oxygen free radicals, which cause vascular endothelial damage, aggravate cerebral ischemic anoxia, and increase the risk of cerebrovascular disease [19, 28, 67, 68]. Therefore, the higher the serum levels of Hcy and hs-CRP, the more severe the cognitive impairment [19, 68–70]. In this study, the serum Hcy and hs-CRP levels decreased significantly in the GLJC group after treatment, which indicates that GLJC may improve VaCI by inhibiting oxidation and inflammatory reactions.

In TCM, VaMCI is closely related to a deficiency in kidney essence, which leads to a series of syndromes that manifest as symptoms, such as forgetfulness, dizziness, fatigue, tinnitus, and soreness and weakness of the waist and knees. Therefore, we assessed the improvement of TCM syndromes in patients with VaMCI using the CM-SS. We found that the GLJC group exhibited significant improvement in the symptoms of intelligence decline, soreness and weakness of the waist and knees, and burnout compared with the Ginkgo group. This demonstrates a significant clinical effect of GLJC for improving the TCM syndromes of patients with VaMCI. These results suggest that GLJC can not only fight aging but also improve VaMCI [40]. This finding further expands the clinical efficacy of GLJC further. However, the sample size of this study is limited. Thus, more clinical and basic studies are needed to clarify the role of GLJC in improving cognitive impairment.

This study has several limitations. First, the sample size is small and lacks universality. Second, more patients dropped out in the GLJC group, possibly, because they did not benefit significantly from the intervention or due to poor patient compliance. Third, the control for confounding factors was not comprehensive enough. For example, factors, such as daily lifestyle habits, dietary habits, and physical activity, were not considered. Finally, the use of multiple evaluators may have resulted in subjective differences.

## 5. Conclusion

The study showed that GLJC can improve the cholinergic system and inhibit inflammatory reactions, which may help protect vascular endothelial cells and improve cognitive function in patients with VaMCI. Furthermore, the results showed that this treatment can improve symptoms of TCM syndromes in patients with VaMCI.

## Figures and Tables

**Table 1:** Baseline characteristics of the patients.

	GLJC group (*n* = 37)	Ginkgo group (*n* = 45)	*P* value
Age, years, mean (SD)	69.54 (7.10)	70.58 (7.22)	0.516
Sex, *n* (%)			0.204
Male	20 (54.95)	18 (40.00)	
Female	17 (45.95)	27 (60.00)	
Education level, *n* (%)			0.869
Below primary school	0 (0)	0 (0)	
Primary school	6 (16.22)	6 (13.33)	
Middle school	16 (43.24)	18 (40.00)	
High school or above	15 (40.54)	21(46.67)	
BMI, kg/m^2^, median (IQR)	23.42 (22.20–24.97)	23.53 (21.81–25.32)	0.783
Risk factors, *n* (%)			0.616
Hypertension	16 (43.24)	15 (33.33)	
Diabetes	12 (32.43)	9 (20.00)	
Hyperlipidemia	4 (10.81)	8 (17.78)	
Coronary artery disease	11 (29.73)	11 (24.44)	
Abnormally craniocerebral CT, *n* (%)			0.370
Cerebral infarction	21 (56.76)	31 (68.89)	
Cerebral ischemia	9 (24.32)	7 (15.56)	
Leukodystrophy	10 (27.03)	8 (17.78)	

Note: sex, education level, hypertension, diabetes, hyperlipidemia, coronary heart disease history, and abnormal craniocerebral CT scans were analyzed using the chi-square test (*χ*^2^ test). BMI: body mass index; CT: computed tomography.

**Table 2:** Comparison of scale scores and serum indexes before treatment in the two groups.

Scale	GLJC group (*n* = 37)	Ginkgo group (*n* = 45)	*P* value
MoCA, mean (SD)	20 (2.43)	20.47 (2.90)	0.438
MMSE, median (IQR)	24 (24-25)	24 (24-25)	0.578
ADAS-Cog, mean (SD)	21.54 (7.30)	20.63 (9.08)	0.624
CM-SS, mean (SD)	19.84 (5.65)	20 (4.83)	0.889
HIS, median (IQR)	9 (8–10.5)	9 (8–11)	0.985
ADL, median (IQR)	23 (21–25)	23 (22–25)	0.921
CDR, median (IQR)	0.5 (0–0.5)	0 (0–0.5)	0.307
Ach (pmol/mL), median (IQR)	135.63 (95.32–224.60)	130.84 (85.84–200.94)	0.783
AchE (pg/mL), median (IQR)	291.85 (264.54–348.78)	305.24 (270.62–371.81)	0.418
Hcy (*µ*mol/L), mean (SD)	12.62 (3.06)	11.80 (2.57)	0.187
hs-CRP (mg), median (IQR)	3.59 (1.92–4.98)	3.50 (2.55–5.17)	0.830

Note: the normally distributed MoCA, ADAS-Cog, and CM-SS scores and the Hcy levels are expressed as mean (SD). The nonnormally distributed variables are expressed as median (IQR).

**Table 3:** Comparison of MoCA, MMSE, and ADAS-Cog scores after treatment in the two groups.

		GLJC group (*n* = 37)	Ginkgo group (*n* = 45)	*P* value
MoCA	Before treatment	20 (2.43)	20.47 (2.90)	0.438
	12-week treatment	23 (22–24)	22 (21–24)	0.180
	24-week treatment	25 (23.5–26)	23 (22.5–24)	≤0.001^*∗*^
MMSE	Before treatment	24 (24-25)	24 (24-25)	0.578
	12-week treatment	26 (25–28)	26 (25–27)	0.663
	24-week treatment	28 (27–29)	27 (25–28)	0.044^*∗*^
ADAS-Cog	Before treatment	21.54 (7.3)	20.63 (9.08)	0.624
	12-week treatment	15.82 (7.81)	15.76 (8.28)	0.972
	24-week treatment	12.02 (5.98)	14.02 (7.59)	0.185

Note: ^*∗*^significant effect (*P* < 0.05). Normally distributed data are expressed as mean (SD), whereas nonnormally distributed data are expressed as median (IQR).

**Table 4:** Comparison of a single item in the MoCA scale after treatment in the two groups.

Item	GLJC group (*n* = 37)		Ginkgo group (*n* = 45)		*P* value
	Before treatment	After 24-week treatment	Before treatment	After 24-week treatment	
Visual space and execution	3 (2–4)	5 (4–5)	3 (2–4)	3 (3–5)	0.001^*∗*^
Naming	3 (2–3)	3 (3–3)	3 (2–3)	3 (3–3)	0.864
Attention	4 (3–5)	5 (4–6)	4 (3–5.5)	5 (4–6)	0.936
Language	2 (1–3)	2 (2–3)	2 (1–3)	2 (2–3)	0.171
Abstraction	1 (1–2)	2 (1–2)	1 (1–2)	2 (1–2)	0.661
Delayed recall	2 (1–2)	3 (1.5–4)	2 (1–2.5)	2 (1–3)	0.194
Orientation	5 (4–5.5)	6 (5–6)	5 (4–6)	5 (4.5–6)	0.015^*∗*^

Note: ^*∗*^significant effect (*P* < 0.05). Data are expressed as median (IQR).

**Table 5:** Comparison of serum Ach, AchE, Hcy, and hs-CRP levels after treatment in the two groups.

Index		GLJC group (*n* = 37)	Ginkgo group (*n* = 45)	*P* value
Ach (pg/mL)	Before treatment	135.63 (95.32–224.60)	130.84 (85.84–200.94)	0.783
	24-week treatment	258.35 (236.61–304.61)	230.74 (191.10–284.93)	0.007^*∗*^
AchE (pg/mL)	Before treatment	291.85 (264.54–348.78)	305.24 (270.62–371.81)	0.418
	24-week treatment	242.39 (204.79–279.07)	251.40 (233.75–275.45)	0.222
Hcy (*μ*mol/L)	Before treatment	12.62 (3.06)	11.80 (2.57)	0.187
	24-week treatment	5.82 (1.26)	6.49 (1.13)	0.013^*∗*^
hs-CRP (mg/L)	Before treatment	3.59 (1.92–4.98)	3.50 (2.55–5.17)	0.830
	24-week treatment	0.76 (0.33–1.72)	0.57 (0.31–1.68)	0.867

Note: ^*∗*^significant effect (*P* < 0.05). Normally distributed data are expressed as mean (SD), whereas nonnormally distributed data are expressed as median (IQR).

**Table 6:** Comparison of total CM-SS scores in the two groups before and after treatment.

	GLJC group (*n* = 37)	Ginkgo group (*n* = 45)	*P* value
Before treatment	20.22 (5.41)	20 (4.83)	0.849
12-week treatment	14 (12–17)	17 (15–20.5)	0.003^*∗*^
24-week treatment	12 (10–15)	14 (11.5–16)	0.013^*∗*^

Note: ^*∗*^significant effect (*P* < 0.05). Normally distributed data are expressed as mean (SD), whereas nonnormally distributed data are expressed as median (IQR).

**Table 7:** Comparison of the overall efficacy rate of the CM-SS after treatment.

	GLJC group (*n* = 37)	Ginkgo group (*n* = 45)	*P* value
Ineffective cases	12	24	0.075
Improved cases	24	21	
Effective cases	1	0	
Cured cases	0	0	

**Table 8:** Comparison of the efficacy of a single item in the CM-SS after treatment in the two groups.

Symptom	GLJC group (*n* = 37)		Ginkgo group (*n* = 45)		*P* value
	Effective cases (*n*)	Efficacy rate (%)	Effective cases (*n*)	Efficacy rate (%)	
Forgetfulness	18	48.65	13	28.89	0.066
Intelligence decline	14	37.84	2	4.44	≤0.001^*∗*^
Soreness and weakness of the waist and knees	25	67.57	17	37.78	0.007^*∗*^
Burnout lie	29	78.38	22	48.89	0.006^*∗*^
Grim and silence	9	24.32	25	55.56	0.004^*∗*^
Panic	9	24.32	16	35.56	0.272
Dizziness	18	48.65	25	55.56	0.533
Tinnitus	17	45.95	22	48.89	0.791
Acousma	6	16.22	7	15.56	0.935
Flushed cheeks	9	24.32	16	35.56	0.272
Tongue-coating	18	48.65	16	35.56	0.231
Pulse condition	19	51.35	14	31.11	0.063

Note: ^*∗*^significant effect (*P* < 0.05).

## Data Availability

The data set used and analyzed during the current study is available from the corresponding author Lina Ma (Email malina19860814@126.com) or Hao Li (Email xyhplihao1965@126.com) on reasonable request.
